# The Kennedy Matter

**Published:** 1892-07

**Authors:** 


### Society Notes.

For Daniel's Texas Medical Journal.

### THE KENNEDY MATTER.

### West Texas Medical Society—Official Letter to the Secretary of the Texas State Medical Association.

SAN ANTONIO, TEXAS, June 1, 1892.

*H. A. West, M. D., Secretary Texas State Medical Association:*

DEAR SIR:—We are instructed by the West Texas Medical

---

NOTE—The above letter is published at the request of West Texas Medical Society, of which Society the JOURNAL is the official organ.

Association to endeavor to correct a very serious misapprehension on the part of the Judicial Council, and in which you seem to share, in the matter of the expulsion of Dr. James Kennedy from the West Texas Medical Association.

Doctor Kennedy was expelled for a grossly insulting communication addressed by him to the Board of Censors in reply to a courteous request that he would explain the appearance of an advertising paragraph in relation to a case and surgical operation, in a daily newspaper. This was followed by a still more insulting article in the same daily paper, which he fully avowed. For this, and for this only, Dr. Kennedy was, and will remain, expelled.

The documents relating to this matter were transmitted to you for the information of the Texas State Medical Association, not for revision or criticism, no case being before the Judicial Council, no appeal having been taken by Dr. Kennedy, as he himself informed the Judicial Council, and having sought redress in a civil court, his expulsion was, and is, a finality. See By-Laws, Article XII., Section 2, as follows: "All questions based upon real or supposed professional or personal grievances must be adjudicated by the county or district societies of which the parties may be members, and it is hereby declared that all such questions are beyond the jurisdiction of the Judicial Council, *provided no appeal from the lower Council appears.*"

At this point we are regretfully instructed to call your attention to your own singular action in the premises. In transmitting these documents to the Judicial Council, they were accompanied by a letter which you claim to have been private, though how an official act should be performed through a private letter, is not clear to the common sense of this Association, and this *letter* was not returned with the papers relating to this affair, as appears from your letter to the Secretary of the West Texas Medical Association of the date of May 18th. In this letter of transmittal of a private nature, you stated that to your mind the West Texas Medical Association had failed to make out its case. The West Texas Medical Association respectfully but urgently requests that you will inform them what case was before the Judicial Council? Your whole course in the matter, as shown by your own letters, savors of an attempt to forestall and prejudice the opinion of a judicial body in the discharge of its judicial functions.

Another point to which we are instructed to call your attention is the fact that the affidavit sworn to by Drs. Cross and Young was never returned to this Association, and was consequently abstracted from the record. The existence of this document is established by the letter of the chairman of the Judicial Council now in the possession of this Association.

In conclusion, we are instructed to state that to the apprehension of the West Texas Medical Association the Judicial Council of the Texas State Medical Association had no jurisdiction, having no case before it, and that the action of a few individual members of the Texas State Medical Association can have no judicial weight with the West Texas Medical Association, and said Association respectfully declines to correct irregularities of the existence of which it is entirely ignorant.

And now, doctor, to prevent any misapprehension as to the *official* character of this communication, the President and Secretary of the West Texas Medical Association are instructed to sign and seal it, and to request that you communicate it to the President of the Texas State Medical Association.

We have the honor to be, your obedient servants,

[L. S.] B. F. KINGSLEY, M. D.,
President W. T. M. A.
D. BERREY, M. D.,
Secretary W. T. M. A.

---

The following, as apropos of the letter from the West Texas Medical Association, is produced at request of Dr. West:

REPORT OF JUDICIAL COUNCIL.

In the matter of the expulsion of Dr. James Kennedy from the West Texas Medical Association, it was resolved that the papers submitted disclose irregularities and non-conformities to their By-Laws and Constitution of such a gross character that we cannot approve them without doing an injustice to Dr. Kennedy; therefore we order that they be returned to the West Texas Medical Association for correction and legal adjudication at home before appealing to the Texas State Medical Association, and that is Dr. Kennedy's proper course, after trial by the local Association.

[Signed] J. C. LOGGINS, M. D., Chairman.

